# Repurposing a psychoactive drug for children with cancer: *p27*^*Kip1*^-dependent inhibition of metastatic neuroblastomas by Prozac

**DOI:** 10.1038/s41389-019-0186-3

**Published:** 2020-01-02

**Authors:** Sandra Bibbo’, Alessia Lamolinara, Emily Capone, Stefania Purgato, Alexia Tsakaneli, Valeria Panella, Michele Sallese, Cosmo Rossi, Paolo Ciufici, Valentina Nieddu, Vincenzo De Laurenzi, Manuela Iezzi, Giovanni Perini, Gianluca Sala, Arturo Sala

**Affiliations:** 1 Dipartimento di Scienze Psicologiche, della Salute e del Territorio, Università “G D’Annunzio” Chieti-Pescara, 66100 Chieti, Italy; 2 Centro di Studi e Tecnologie Avanzate (CAST), Università “G D’Annunzio” Chieti-Pescara, 66100 Chieti, Italy; 3 Dipartimento di Medicina e Scienze dell’Invecchiamento, Università “G D’Annunzio” Chieti-Pescara, 66100 Chieti, Italy; 4 Dipartmento di Scienze Mediche, Orali e Biotecnologiche, Università “G D’Annunzio” Chieti-Pescara, 66100 Chieti, Italy; 5 Dipartimento di Farmacia e Biotecnologia, Universita’ di Bologna, 40126 Bologna, Italy; 6 Institute of Environment, Health and Societies, College of Health and Life Sciences, Brunel University London, UB8 3PH Uxbridge, UK; 7 Present Address: Unit of Gynecological Oncology Research, European Institute of Oncology, Via G. Ripamonti 435, 20141 Milano, Italy

**Keywords:** Paediatric cancer, Targeted therapies, Oncogenes

## Abstract

The MYC family of transcription factors is a major driver of human cancer and potential therapeutic target. However, no clinically viable drugs have been yet developed that are able to directly tackle MYC oncoproteins. In our laboratory, we are exploring alternative approaches aiming to disturb signalling downstream of MYC. MYCN is frequently activated in neuroblastoma, a paediatric solid malignancy that, in its metastatic form, has a very poor prognosis. An important pathway regulated by MYC is the CKS1/SKP2/p27^kip1^ axis. In this study, we have repurposed the anti-psychotic drug Prozac to disrupt CKS1/SKP2/p27^Kip1^ signalling and assess its potential as an anti-neuroblastoma agent in vitro and in vivo. Using DNA editing technology, we show that stabilisation of p27^Kip1^ operated by Prozac in MYC-activated cells is essential for the anti-neuroblastoma activity of the drug. Furthermore, dosing mice with a concentration of Prozac equivalent to that used in long-term clinical trials in children with psychiatric disorders caused a significant reduction of metastatic disease in two models of high-risk neuroblastoma. The favourable toxicity profile of Prozac suggests that long-term treatments might be implemented in children with MYC/CKS1^high^ neuroblastomas.

## Introduction

Neuroblastoma is a rare cancer of the sympathetic nervous system that occurs during early childhood and infancy. Although it only accounts for 7% of cancer diagnosis of children under 15 year, it is responsible for most paediatric mortalities owing to solid tumours and is the commonest cancer diagnosed within the first year of life^1^. Neuroblastomas arise from sympathoadrenal lineage neural crest cells and primary tumours form in the sympathetic nervous system, usually within the adrenal medulla or paraspinal ganglia^2^. A major molecular alteration in neuroblastoma is the amplification of the locus encoding the oncogenic transcription factor MYCN. MYCN belongs to a small gene family that includes c-MYC and l-MYC, and encodes for a 60–64 kDa, nuclear, phosphoprotein, which is one of the BHLH transcription factors responsible for assisting in the differentiation of neuronal progenitor cells transitioning from the neural crest to the sympathetic nervous system^3^. In the nervous system, MYCN has been shown to play a significant role in the development of tissues and the differentiation pathways of neuronal progenitor cells^3^. In neuroblastoma, MYCN amplification is used as a prognostic marker and is indicative of a high-risk disease^1,2^. One of the signalling pathways downstream of MYC proteins is the CKS1/SKP2/p27^Kip1^ axis. When p27^Kip1^ (encoded by *CDKN1B*) is expressed, the complexes formed by the cyclin directed kinase CDK2 cannot be established, and the cell cannot enter the cell cycle. CKS1 (encoded by *CKS1B*) facilitates S phase entry through the degradation of the CDK2 inhibitor, p27^Kip1^^4^. CKS1 acts as a co-factor in the SKP2-cullin-F-box complex for p27^Kip1^ ubiquitination. Although SKP2 binding and subsequent ubiquitination of p27^Kip1^ can occur without CKS1, it is drastically reduced without the presence of CKS1^4,5^. Without CKS1 to bridge the leucine-rich-repeats, SKP2 cannot obtain optimal substrate positioning^6,7^. Although CKS2 shares with CKS1 a similar CDK interaction domain and binding sites for p27^Kip1^, it cannot interact with SKP2 and thereby cannot help reduce p27^Kip1^ levels but, instead, protects it from degradation by SKP2^6,8^. CKS1 recognises phosphorylated p27^Kip1^ through an anion pocket and will bind to promote SKP2 binding, as CKS1 contains specific residues in the N-terminus that allows association with SKP2. CKS2 also contains the anion pocket to recognise and bind to phosphorylated p27^Kip1^ but lacks the association region for SKP2. Therefore, CKS2 protects p27^Kip1^ from degradation, whereas CKS1 promotes degradation, and p27^Kip1^ levels are determined, in part, by the CKS which has the highest concentration^8^. Neuroblastoma tumours have been shown to express high levels of SKP2^9^ and c-MYC upregulates SKP2 expression^10^. Further studies have additionally shown that in neuroblastoma cells MYCN targets a separate E-box location and, similarly to c-MYC, augments transcription of SKP2^11^. Furthermore, c-MYC and MYCN induce *CKS1B* transcription^10,12^. Targeting SKP2 causes p53-independent apoptosis in non-amplified neuroblastoma cells, whereas in MYCN-amplified cells it was noted a decrease in growth but not apoptosis^11^. Likewise, when *CKS1B* was inhibited in tumour cells, apoptosis and growth arrest followed stabilisation of p27^Kip1^^13,14^. A genome-wide, drop-out shRNA screen carried out in our laboratory has identified *CKS1B* as a potential therapeutic target gene in MYCN-amplified neuroblastoma by inducing synthetic lethality^12^. Although pharmacological inhibitors of SKP2 are not currently available^15^, CKS1 can be inhibited by a small molecule that is safe and available. Fluoxetine, also known as Prozac, is a serotonin uptake inhibitor originally developed to treat depression. However, Prozac also has been shown to induce G1 arrest through inhibition of the CKS1–SKP2 binding interaction site, resulting in elevated p27^Kip1^ levels and differentiation of neuronal stem cells^13,16^.

In this study, we investigated whether Prozac could be used to induce stabilisation of p27^Kip1^ and growth arrest/apoptosis of MYC-expressing neuroblastoma cells in vitro and in vivo.

## Results and discussion

### The CKS1 inhibitor Prozac increases p27^Kip1^ expression in neuroblastoma cell lines

**Fig. 1:**
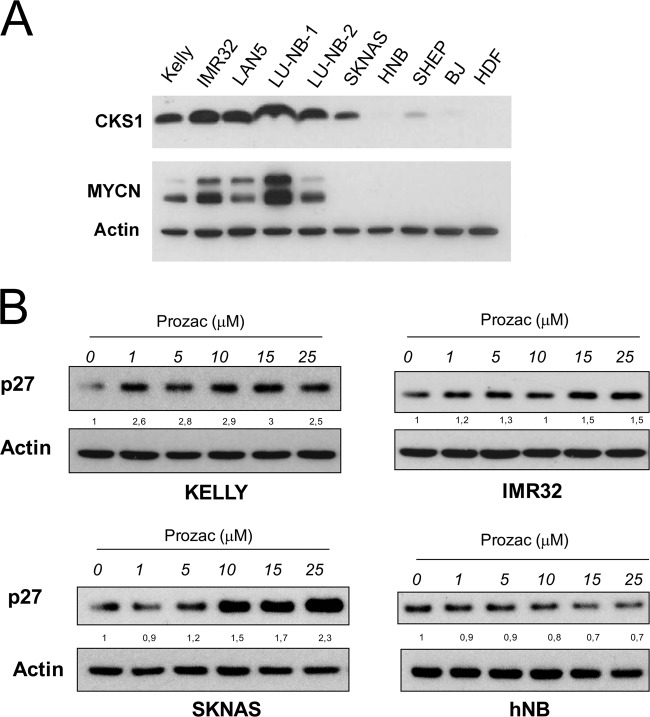
CKS1 and p27 expression in neuroblastoma cell lines. **a** Protein extracts from neuroblastoma cell lines (MYCN amplified = Kelly, IMR32, LAN5, LU-NB-1, LU-NB-2; non-MYCN amplified = SKNAS, SHEP, hNB), normal human dermal fibroblasts (hDF) or immortalised, non-tumourigenic, human fibroblasts (BJ) were subjected to western blot analysis with the indicated antibodies. **b** The selected neuroblastoma cell lines were cultured in the presence of increasing concentrations of Prozac and subjected to western blot analysis with a p27 antibody. Folds of p27 inductions relative to actin are indicated between the blots. Cells were lysed in RIPA Buffer (50 mM Tris-HCl, 1% NP40, 0.1% SDS, 150 mM NaCl) supplemented with protease inhibitor cocktail (Sigma-Aldrich) and phosphatase inhibitor cocktail (Roche) for 30 min in ice. Insoluble material was removed by centrifugation (13,000 rpm for 20 min at 4 °C) and protein concentration was assessed by the method of Bradford. Equal amounts of protein were separated by SDS/PAGE on 15% polyacrylamide gel and transferred into nitrocellulose membrane. Membranes were blocked with 5% non-fat dry milk in PBS 0.1% Tween 20 for 1 h at room temperature and incubated with primary antibodies. The antibodies used were: N-Myc (sc-53993, Santa Cruz Biotechnology, 1:500 dilution), CKS1 (36-6800, Invitrogen 1:400 dilution), β-Actin (A5441, Sigma-Aldrich 1:40000 dilution), p27^Kip1^ (sc-1641, Santa Cruz Biotechnology 1:200 dilution). After washes, membranes were hybridised with appropriate horseradish peroxidase-conjugated secondary antibodies (rabbit and mouse). Detection was performed with Plus-ECL chemiluminescence kit (Bio-Rad, Hercules, CA, USA).

We monitored CKS1 protein levels in a panel of neuroblastoma cell lines with or without activated MYC. As expected, CKS1 levels were higher in MYCN amplified (Kelly, Lan5, LU-NB-1, LU-NB-2) than non-MYCN amplified (hNB, SHEP) neuroblastoma cell lines or normal human fibroblasts (BJ, HDF) (Fig. 1a). It must be noted that non-MYCN-amplified SK-NA-S cells have a mutation that results in activation of c-MYC, which explains the elevated CKS1 levels^17^.

Inhibition of *CKS1B* is synthetically lethal with *MYCN* amplification/overexpression in neuroblastoma cells, suggesting that it may be used to target specifically MYC^high^ tumours^12^. As RNA interference is not yet a viable option in cancer therapy, we used Prozac to disrupt the CKS1–SKP2 interaction, with the aim of causing stabilisation of the product of the tumour suppressor gene *CDKN1B -P27*^*Kip1*^*-*, in neuroblastoma cell lines. Thus, we exposed MYC-expressing, or non-expressing, neuroblastoma cell lines to Prozac and quantified p27^Kip1^ levels by western blotting. The MYC-positive cell lines showed a dose-dependent increase of p27^Kip1^ levels after exposure to the drug. Interestingly, p27^Kip1^ levels did not change in the non-MYCN-amplified cell line hNB (Fig. 1b). This result is consistent with the low levels of CKS1 expression in hNB (Fig. 1a). Prozac is likely to act at the protein level; in the presence of the drug, p27^Kip1^ was stabilised after block of protein synthesis operated by cycloheximide (Supplementary Fig. 1).

### Prozac inhibits short and long-term growth of neuroblastoma cell lines in vitro

**Fig. 2:**
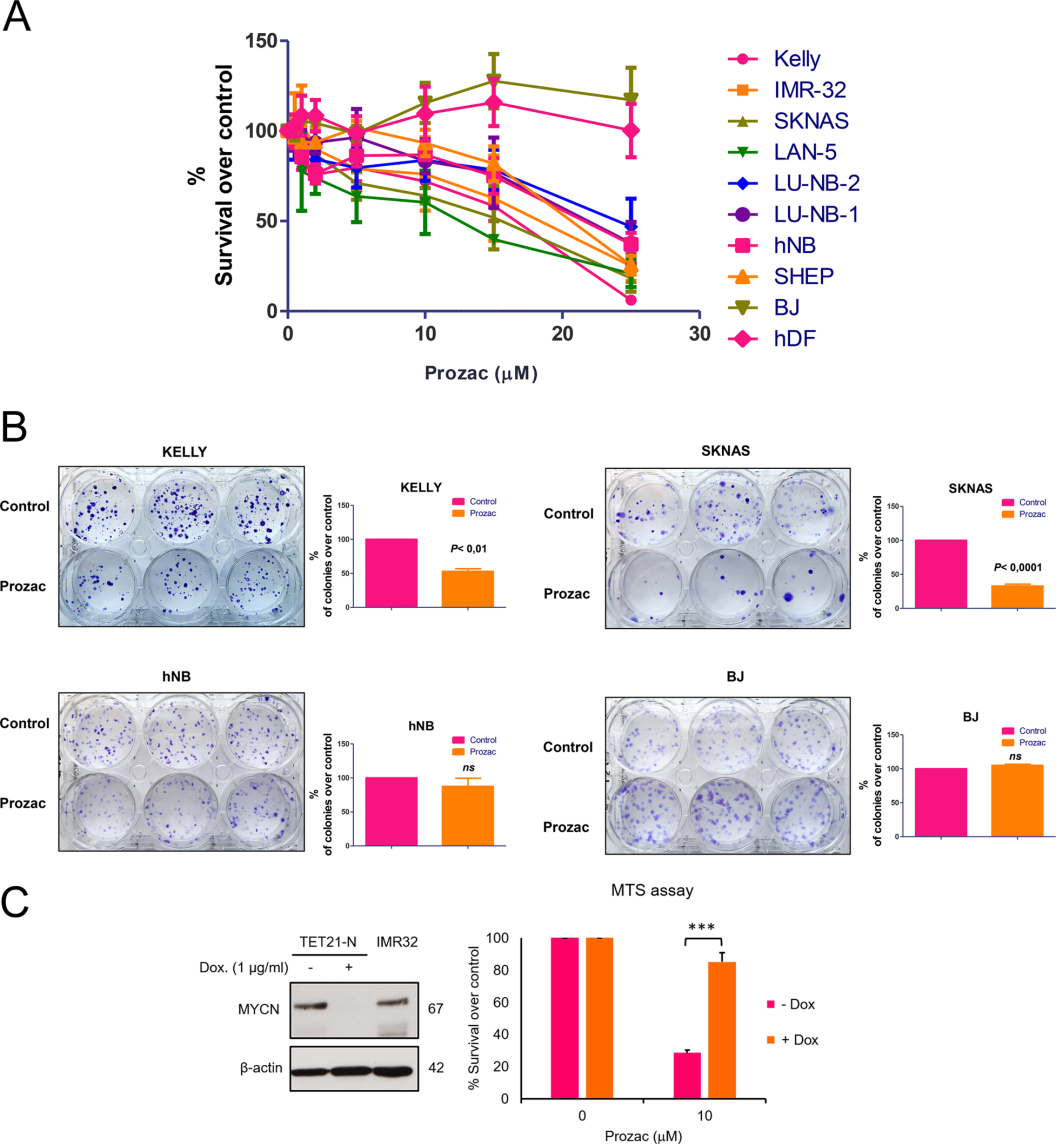
Prozac causes short and long-term inhibition of neuroblastoma cell lines. **a** MTS assays. The MYCN amplified (Kelly IMR32, LAN5, LU-NB-1, LU-NB2), non-amplified (SHEP, SKNAS, hNB) neuroblastoma cell lines were exposed to increasing concentrations of Prozac for 72 h. Normal human dermal fibroblasts (hDF) or immortalised, non-tumourigenic human fibroblasts (BJ) were also exposed to Prozac and served as controls. Each point indicates the average value of four experiments ± SEM. **b** Colony assays. Kelly and SKNAS cells were plated and cultured in the presence or absence of Prozac (10 mM) and the emerging colonies were visualised after 3 weeks in culture with crystal violet and counted. Bars indicate average colony numbers from three independent experiments each performed in triplicate. Error bars indicate SEM. The patient-derived cells LU-NB-1, LU-NB-2 have been established and characterised in the laboratory of Daniel Bexell as described previously^24,25^. hNB cells were isolated from a tumour metastasised in the neck of a 3-year-old male patient in 2011^26^. Cell lines SK-N-AS, IMR32, Kelly, SHEP, LA-N-5 were obtained from the American Type Culture Collection (Teddington, Middlesex, UK). All cell lines were cultured <3 months after resuscitation. The cells were cultured according to manufacturer’s instructions, using a medium supplemented with 10% heat-inactivated fetal bovine serum (FBS; Invitrogen), l-glutamine, 100 units/ml penicillin, and 100 μg/ml streptomycin (Sigma-Aldrich Corporation, St. Louis, MO, USA), and incubated at 37 °C in humidified air with 5% CO_2_. MTT [3-(4,5-dimethyldiazol-2-yl)-2,5-diphenyl tetrazolium bromide] (Sigma-Aldrich). Cells were seeded into 96-well plates at a density ranging from 3,5 × 10^3^ to 5 × 10^3^ cells/well in 200 μl of complete culture medium in the presence or absence of Fluoxetine (Fluoxetine hydrocloride, sc-201125, Santa Cruz Biotechnology). At the end of the experiment, cells were incubated with 100 μl of MTT solution (0.5 mg/ml of MTT in serum free medium) for 2 h at 37 °C. After removal of the MTT solution, cells were incubated with 100 μl of dimethyl sulfoxide (DMSO) for 10 min and the optical density was measured at 570 nm using a multi-plate reader. All experiments were performed in triplicate. For colony forming assays, cells were seeded into six-well plates at a density ranging from 1 × 10^2^ to 8 × 10^2^ cells/well. Cells were left to attach for 48 h, after which 10 μM Fluoxetine was added three times per week. At the end of the experiment, colonies were washed twice with cold PBS, fixed with cold methanol for 10 min in ice and then stained with crystal violet 0.5% for 10 min at room temperature. Colonies were stained after 3 (Kelly, SKNAS) or 2 (BJ, hNB) weeks. Only colonies containing >50 cells were counted. All data are expressed as means ± SE. Statistical significance between different test conditions was determined using Student *t* test. Probability values <0.05 were considered significant. **c** Prozac inhibits neuroblastoma cells in a MYCN-dependent manner. Left panel; western blot analysis showing the expression of MYCN in the presence (MYCN off) or absence (MYCN on) of doxycycline. Beta actin was used as loading control. Antibody used were: MYCN antibody, Santa Cruz sc-53993, dilution 1:200; β-actin, Cell Signaling, dilution 1:1000. Right panel; quantification of metabolic activity (MTS assay) of TET21N cells expressing (−Dox) or non-expressing MYCN (+Dox) in the presence of Prozac. *** Student *t* test *p* *<* 0.001 *n* = 3.

To investigate the therapeutic potential of Prozac in neuroblastoma, we exposed multiple cell lines to increasing concentrations of Prozac for 72 h and assess metabolic activity by MTT assays. As controls, we used normal and immortalised, but non-tumourigenic, human fibroblasts. All neuroblastoma cell lines were inhibited by Prozac, with the majority of the MYC-positive cell lines being slightly more sensitive to the drug. As expected, normal fibroblasts were completely resistant up to the maximum concentration of Prozac used (Fig. 2a). To determine the long-term effects of Prozac, we implemented colony assays in which the neuroblastoma, or normal, cells were exposed to a relatively low concentration of Prozac (10 μM) for 2–3 weeks, after which colonies were scored. There was a clear-cut reduction in the number of colonies formed by the CKS1/MYC^high^ cell lines, whereas the CKS1/MYC^low^ cells (SHEP, hNB, and human fibroblasts) formed colonies normally in the presence of Prozac, suggesting that CKS1 and MYC expression could be used as a biomarker to determine susceptibility to Prozac inhibition (Fig. 2b). To further confirm the role of MYC in determining sensitivity of neuroblastoma cells to Prozac, we used a switchable TET-OFF system in which transgenic expression of MYCN can be turned off in the presence of doxycyclin. Proliferation of MYCN expressing SHEP (TET21N) is markedly inhibited by a low concentration of Prozac (10 μM); however, when MYCN expression was ablated they became resistant, confirming the hypothesis that Prozac triggers synthetic lethality in MYC-expressing cells (Fig. 2c).

To gain mechanistic insights into the nature of the inhibition, we assessed cell cycle phases and quantified DNA fragmentation, diagnostic of apoptosis. Prozac did not cause significant changes in cell cycling activity, but it induced a significant increase of DNA fragmentation in the CKS1/MYC^high^ cell lines Kelly and SKNAS compared to CKS1/MYC^low^ hNB and BJ (Supplementary figure 2). The hypothesis that Prozac might cause apoptosis of neuroblastoma cells is corroborated by a recent study showing that the MYCN-amplified SK-N-BE^2^ cell line undergoes apoptosis in the presence of the drug^18^.

### Expression of *CDKN1B* is essential for the antiproliferative effect of Prozac

**Fig. 3:**
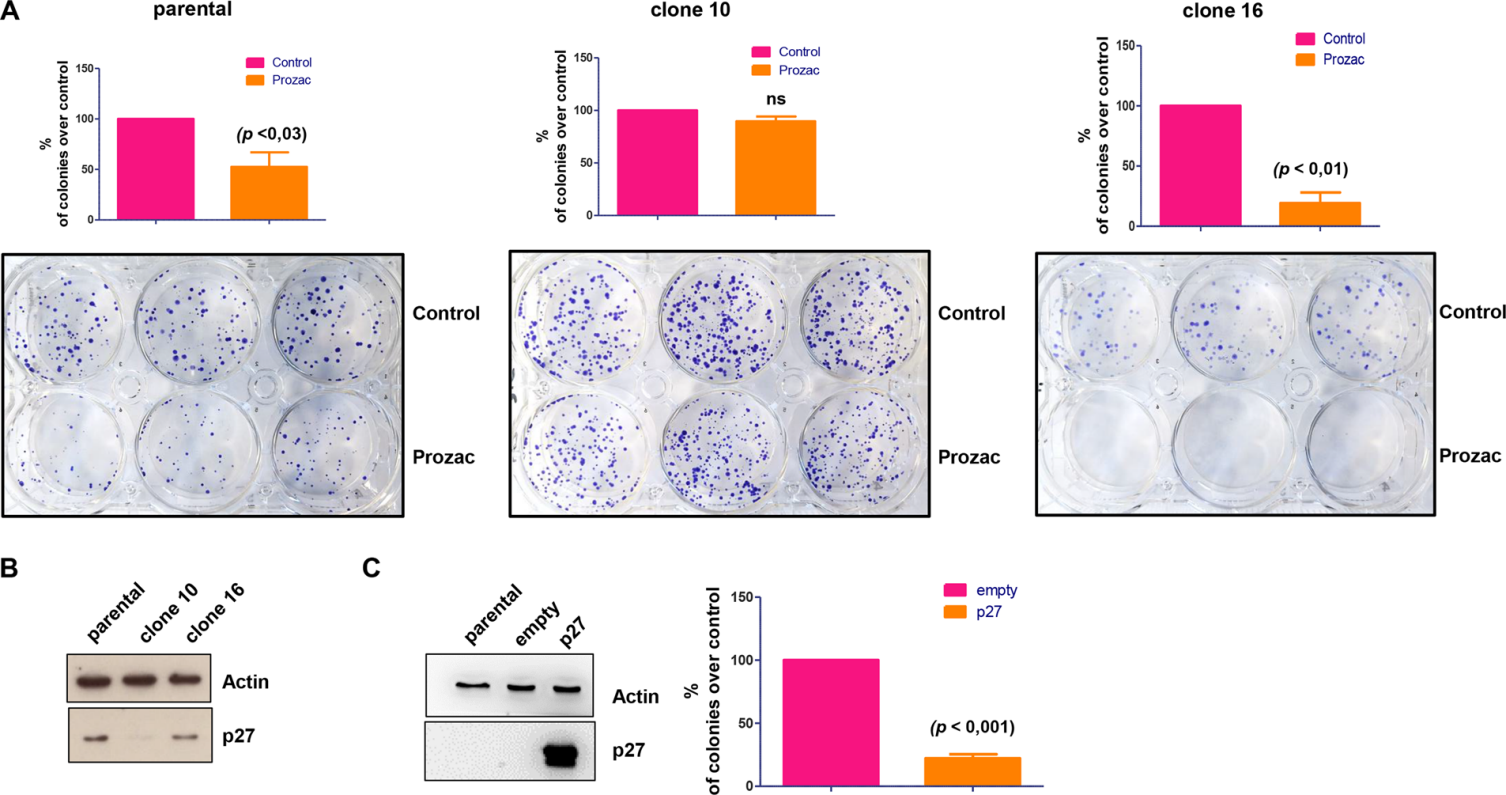
Crispr/cas9 deletion of the *CDKN1B* gene induces resistance to the anticancer effect of Prozac. **a** Parental or crispr/cas9-treated Kelly cell lines were cultured in the presence or absence of Prozac for 3 weeks in colony formation assays. Colonies were counted after staining cells with crystal violet. Bars indicate mean values ± SEM (*n* = 4). Statistical significance between different test conditions was determined using Student *t* test. Probability values < 0.05 were considered significant. A pair of single-guide RNAs (sgRNAs) was used to create two double strand breaks (DSBs) upstream and downstream of the *CDKN1B* locus, in order to delete the intervening DNA segment by non-homologous end joining (NHEJ) repair. The two specific sgRNAs were designed using the BlueHeron Guide RNA Target Design Tool and CasOT software:^27^ upstream sgRNA 5’-CGGCGACCTTCGCGGTCCTC-3’ and downstream sgRNA 5’-CAAAGGGACGTTCACGGCGA-3’. The sgRNAs were cloned into the pSpCas9-2A-GFP (PX458) vector (Addgene) and transfected into Kelly cells using Lipofectamine 3000 (Thermo Fisher Scientific). 48 h after transfection, GFP-positive cells were sorted by FACS (S3e™ Cell Sorter, Bio-Rad Laboratories, Inc., California, USA) and were plated individually into 96-well plates by limiting dilution. Clonal cell lines were expanded and gene deletion was tested by PCR, using the following primers: Fw 5’-TAAGTGCCGCGTCTACTCCT-3' and Rv 5’-AGCTTTCGCTGCTTTCTCAG-3'. Gene deletion was confirmed by DNA sequencing (Macrogen Spain, Madrid, Spain). PCR amplification and DNA sequencing of the non-deleted allele were performed using the following primers: Fw 5’-TAAGTGCCGCGTCTACTCCT-3' and Rv 5’-ATACGCCGAAAAGCAAGCTA-3'. **b** Crispr/cas9 targeted Kelly clones 16 and 10 were subjected to western blot analysis with a p27^Kip1^ antibody. An actin antibody was used as loading control. **c** Ectopically overexpressed p27^Kip1^ induces growth arrest of SKNAS cells. Cells were seeded in 60-mm dishes at a density of 1 × 10^6^ cells/dish and transfected with control (pcDNA3) or pcDNA3-p27 vectors using Lipofectamine 2000 (Invitrogen, 11668-019) following manufacturer’s instructions. 72 h after transfections, the cells were used for western blot analysis to verify expression of ectopic levels of p27^Kip1^ (left panel) or plated into a six-well plate at a density of 5000 cells/well and selected with 800 µg/ml G418. 3 weeks later, colonies were fixed with cold methanol, stained with 0.5% crystal violet and quantified (right panel). Bars indicate mean values ± SEM (*n* = 3).

To confirm that stabilisation of p27^Kip1^ levels operated by inhibition of CKS1 is required for the growth inhibiting effect of Prozac, we deleted the *CDKN1B* gene using crispr/Cas9 technology. After clonal expansion of crispr/cas9-treated Kelly cells, we selected a clone (clone 10) with disrupted *CDKN1B* alleles (Supplementary figure 3). As a further control, we expanded another clone (clone 16) that had undergone the crispr/cas9 procedure but retained expression of p27^Kip1^. Next, we implemented colony formation assays in which we exposed the crispr/cas9-treated clones, or the parental Kelly cell line, to Prozac. Notably, the p27^Kip1^-null clone 10 regained the ability to form colonies in the presence of Prozac, whereas the parental cell line Kelly or clone 16, which had retained expression of p27^Kip1^, were sensitive to the drug (Fig. 3a). Western blot analysis confirmed that expression of p27^Kip1^ in clone 10 was reduced almost to zero, whereas clone 16 and parental cells presented similar p27^Kip1^ levels (Fig. 3b). To confirm that Prozac-induced p27^Kip1^ is key for the inhibitory effect of the drug, we ectopically overexpressed p27^Kip1^ in SKNAS cells, which express low basal levels of p27^Kip1^ (Fig. 3c, left panel). Overexpression of p27^Kip1^ caused a marked inhibition of colony formation (Fig. 3c, right panel). Overall, these experiments clearly establish that Prozac inhibits neuroblastoma proliferation via stabilisation of the tumour suppressor p27^Kip1^.

### Prozac inhibits metastatic growth of high-risk neuroblastoma cell lines in vivo

**Fig. 4:**
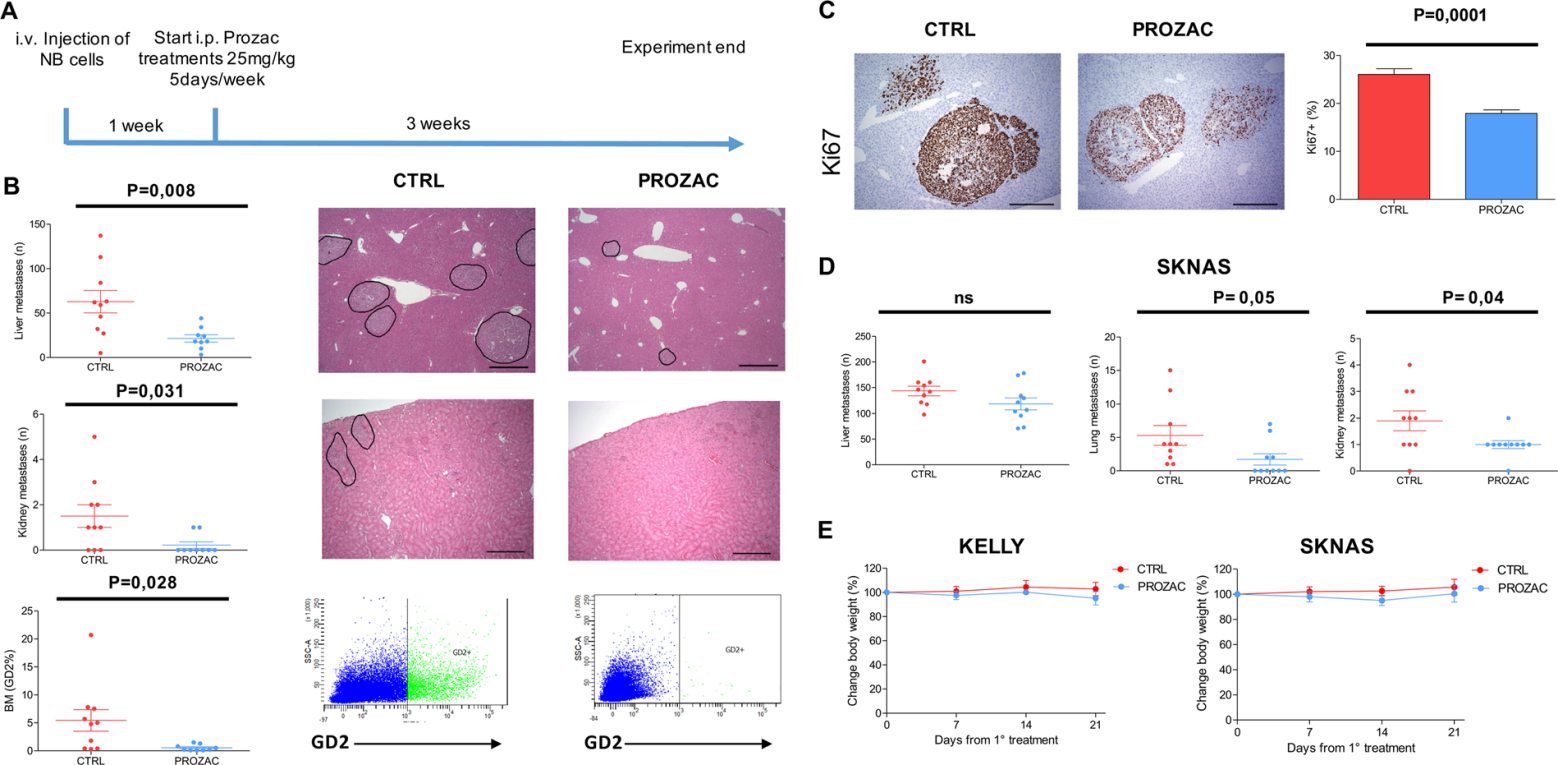
Prozac inhibits metastasis formation and proliferation in mouse models of neuroblastoma. **a** workflow describing the stages of the experiment. **b** Enumeration of KELLY metastases in the indicate organs. Representative sections of livers stained by haematoxylin–eosin are shown in the right of the panel; metastases are evidenced by circles. FACS plots showing GD2 staining in the bone marrow are shown in the bottom of the panel. **c** Representative section of livers containing metastases positive to KI67 staining are shown in the left of the panel. The quantification is in the right. **d** Quantification of SKNAS metastases. **e** Assessment of mice body weight during the course of treatment with Prozac. Error bars indicate mean values ± SEM (*n* = 10). Statistical significance between different test conditions was determined using the Student *t* test. Probability values < 0.05 were considered significant. Immunodeficient Nod Scid Gamma (NSG) mice were purchased from the Jackson Laboratory and bred in the animal facility of CeSI-Met, G. D’Annunzio University, Chieti. Animal care and experimental procedures were approved by the Ethics Committee for Animal Experimentation of the institute according to Italian law (Authorization no. 292/2017-PR and Authorization no. 514-2018/PR). Eight-weeks old female NSG mice (9–10 mice per group) were injected via the lateral tail vein with 5 × 10^5^ KELLY or SKNAS neuroblastoma cells; after a week, mice were randomised into two groups that received vehicle or Prozac (20 mg/Kg) 5 days per week for 3 weeks. The animal health status was monitored daily and body weight was measured every 3–4 days during the treatment. After 28 days of tumour cells injections, mice were killed and organs were harvested, fixed in 10% neutral buffered formalin. To optimise the detection of microscopic metastases and ensure random sampling, lungs, and livers were cut transversally into 2.0 mm thick parallel slabs, resulting in 5–8 slabs for lungs and 6–8 slabs for livers. The slabs were then embedded, cut surface down and sections were stained with Haematoxylin and Eosin. Slides were independently evaluated by two pathologists to quantify the number of tumor lesions in the organs harvested. The major leg bones were harvested for extraction of bone marrow cells, by cutting the edges of the bones and flushing with 1 ml syringes containing PBS through the bone; the cells extracted were stained with a GD2 antibody (clone 14.2Ga; Millipore) for quantification of human neuroblastoma cells by flow cytometry.

Tumour relapse is common in children with high-risk neuroblastoma and finding a drug with a favourable toxicity profile to prolong remission would be a major clinical advance. Prozac could be particularly useful in the context of minimal residual disease, where chemotherapy-resistant neuroblastoma cells in the bone marrow and other organs eventually cause cancer to relapse. To emulate this situation, we have implemented a pseudo-metastatic model in which Kelly (MYCN amplified) and SKNAS (c-MYC mutant) neuroblastoma cells are injected in the tail vein of immunocompromised mice. In these models, tumour cells colonise preferably the liver and kidneys. Kelly cells have also a tropism for the bone marrow, whereas SKNAS cells are unable to infiltrate the bone marrow but can invade lungs. To allow organ seeding and establishment of micro-metastases, treatments with a pharmacological concentration of Prozac was started 1 week after tumour cell injections and continued for 3 weeks (a scheme of the experiment is shown in Fig. 4a). After mice killing, gross metastases in the liver, kidneys, and lungs were quantified by microscopy, and neuroblastoma cells in the bone marrow were enumerated by flow cytometry with a GD2 antibody. In the Kelly model, Prozac treatments caused a sharp, statistically significant reduction of the number of metastases and neuroblastoma cell proliferation, as indicated by reduced to positivity to the ki67 marker, suggesting that the drug is antimetastatic (Fig. 4b, c). The anticancer activity of Prozac was largely confirmed in the SKNAS model, although the decrease in metastases formation in the liver did not reach statistical significance (Fig. 4d). Prozac treatments were very well tolerated by mice, as indicated by stable body weights (Fig. 4e).

In spite of important progresses in the understanding of the molecular alterations underpinning the aggressive behaviour of neuroblastoma, this cancer still poses a formidable challenge to clinicians. Based on the recent discovery of mutations at the level of genes that activate neuroblastoma oncogenic pathways, such as MYCN, ALK, ATRX, TERT^1,2^, some investigational, molecularly targeted drugs are being experimented in clinical trials. However, as of today, children are still treated with highly toxic mixtures of chemotherapeutic drugs, radiation and potentially unsafe compounds such as retinoids. Although immunotherapy with the GD2 antibody has significantly improved the outcome of high-risk patients, more than half of the treated children do not show a clinically relevant response^19^.

We have recently implemented a genome-wide shRNA screen to detect MYCN-dependent vulnerabilities in neuroblastoma. In this study, we identified, among other genes, *CKS1B* as a synthetic lethal partner of MYCN. MYC and CKS1 are involved in a crosstalk with p27^Kip1^; MYC promotes degradation of p27^Kip1^ via activation of CKS1 and the ubiquitin ligase skp2. The latter modulates MYC protein stability, also acting as a transcriptional co-factor and enhancer of MYC-dependent activation of cell cycle-related genes^20^. In the present study, we show that Prozac induces stabilisation of p27^Kip1^ in MYC^high^ neuroblastoma cells, resulting in reduced proliferation and increased apoptosis in vitro. In vivo, Prozac displays a potent antimetastatic activity, suggesting that it may be repurposed to treat cancer patients.

It is important to note that the range of Prozac doses used in clinical trials in children with psychoses is 0.8–2 mg/kg^21,22^, equivalent to 10–24.6 mg/kg in the mouse system, after dose conversion^23^. The drug dosage used in our study, 20 mg/kg, is well within this range. Notably, duration of treatments in one of the human trials were up to 32 months, with manageable neurologic side effects, indicating that long-term treatment of children with anticancer concentrations of Prozac is feasible^21^. As high expression of MYCN and CKS1 mark cells that are susceptible to Prozac in long-term assays in vitro and in vivo, MYCN amplification and elevated CKS1 protein levels in neuroblastoma biopsies could be used as biomarkers to identify patients that might respond to the drug. We conclude that the results presented in this study warrant the opening of clinical trials in which long-term Prozac treatments could be included in consolidation or post-consolidation therapies in patients who are at high risk of disease relapse.

## Supplementary information

Supplementary legends

Figure S1

Figure S2

Figure S3
