# Supplementary material for: Repurposing a psychoactive drug for children with cancer: p27Kip1-dependent inhibition of metastatic neuroblastomas by Prozac
Source: Oncogenesis. 2020 Jan 2;9(1):3. doi: 10.1038/s41389-019-0186-3 (PMC6949307; doi:10.1038/s41389-019-0186-3)
Supplement: Supplementary file 1 — Supplementary legends [file 41389_2019_186_MOESM1_ESM.docx]

Supplementary legends

**Supplementary Figure 1**. P27^Kip1^ is stabilised by Prozac. SKNAS cells (1x10^6^ in 60-mm dishes) were treated simultaneously with 25 µg/ml of cycloheximide (CHX, C-7698, Sigma, Milan, Italy) and 10 µM Fluoxetine for 9h and 24h. The cells were lysed in RIPA Buffer supplemented with protease inhibitor cocktail and phosphatase inhibitor cocktail for 30 min in ice, then equal amounts of protein were subjected to western blot analysis with a p27 antibody (left panel). The normalized densitometric units of p27 are shown in the right panel. Error bars indicate mean values±SEM (n=2).

**Supplementary figure 2.** Propidium iodide DNA staining and FACS analysis. Neuroblastoma (Kelly, SKNAS, hNB) or normal human fibroblasts (BJ) were cultured for 72 hours in the presence or absence of Prozac. Representative cell cycle profiles are shown. Error bars indicate mean values±SEM (n=4). 2×10^6^ cells were harvested and centrifuged for 5 min. 0.5 ml of PBS was added to the cell pellet followed by 70% ethanol to bring the final volume to 5 ml. The cells were stored at 4ºC overnight then washed twice with PBS and resuspended in a solution of propidium iodide (PI, 50 µg/ml) and RNAase (10 µg/ml) which was kept at 4°C overnight in the dark. Cell cycle profiles were quantified using a FACSCantoII flow cytometer (Becton Dickinson, Buccinasco, MI, Italy) and FCSExpress 5 software.

**Supplementary figure 3**. Sequencing of the CDKN1B gene demonstrating crispr/cas9 inactivation of both alleles in clone 10.
