# Supplementary figures and images for: Repurposing a psychoactive drug for children with cancer: p27Kip1-dependent inhibition of metastatic neuroblastomas by Prozac
Source: Oncogenesis. 2020 Jan 2;9(1):3. doi: 10.1038/s41389-019-0186-3 (PMC6949307; doi:10.1038/s41389-019-0186-3)

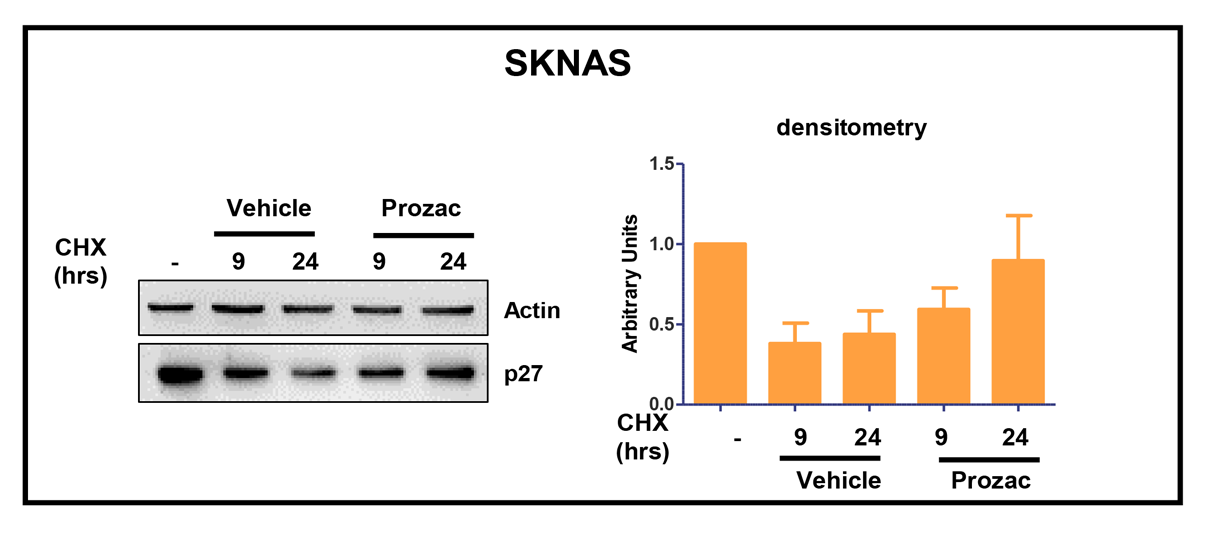

Supplement: Supplementary file 2 — Figure S1 [file 41389_2019_186_MOESM2_ESM.tif]

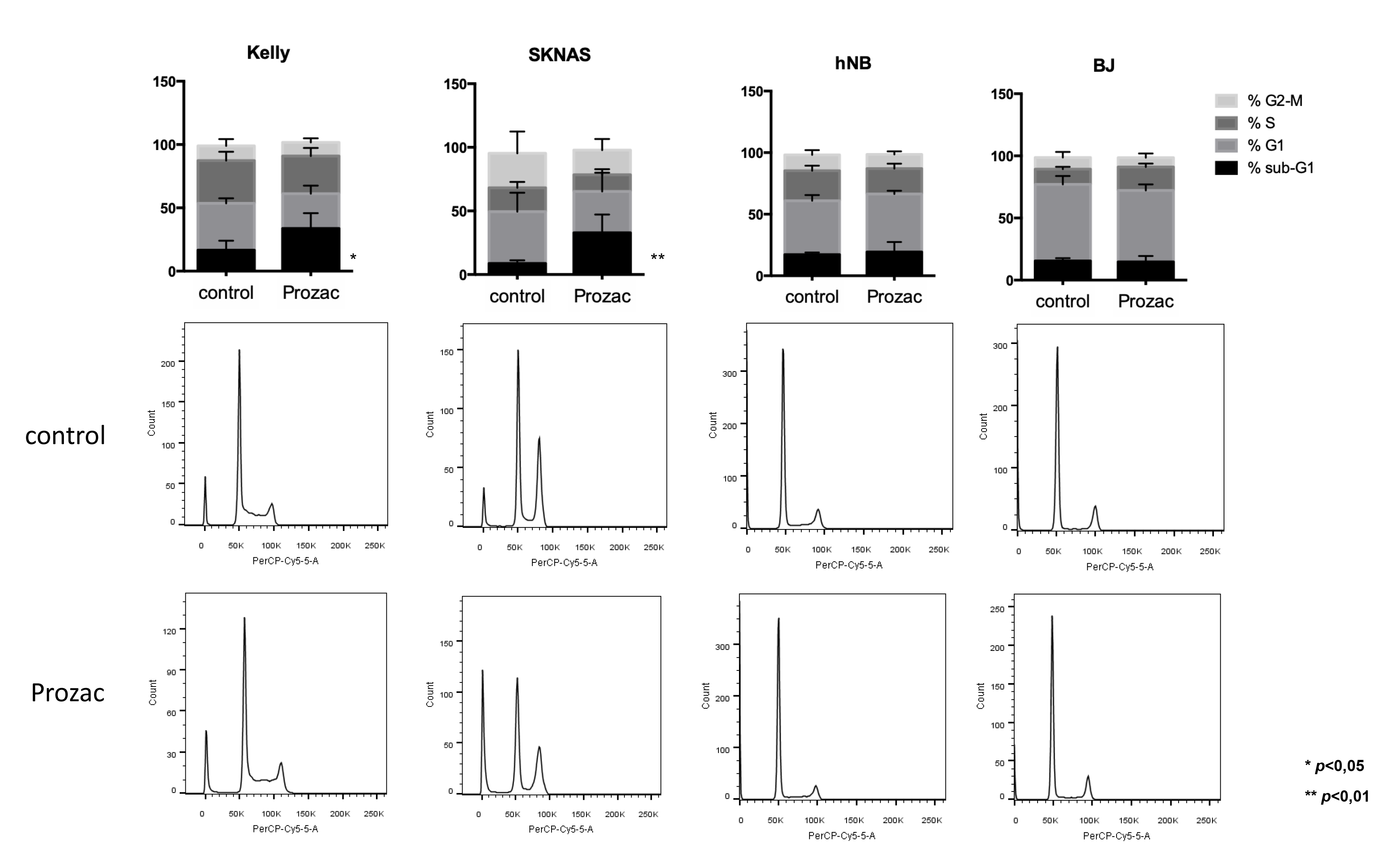

Supplement: Supplementary file 3 — Figure S2 [file 41389_2019_186_MOESM3_ESM.tif]

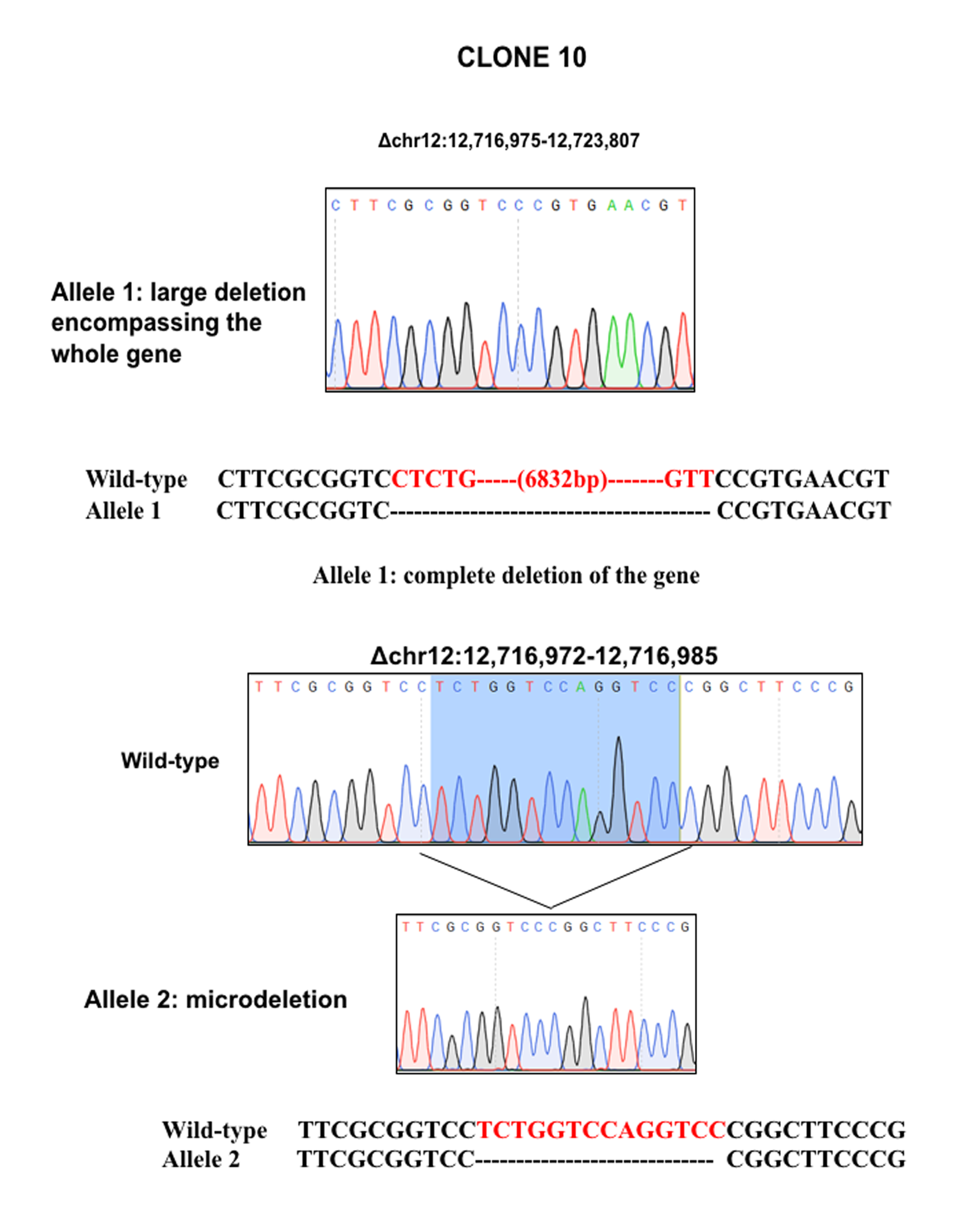

Supplement: Supplementary file 4 — Figure S3 [file 41389_2019_186_MOESM4_ESM.tif]
